# PHYSICAL AND MENTAL HEALTH INFORMATION-SEEKING BEHAVIORS AMONG US-BASED LATINO ADULTS AGES 50–86

**DOI:** 10.1093/geroni/igad104.2073

**Published:** 2023-12-21

**Authors:** Melissa DuPont-Reyes, Alice Villatoro, Elizabeth Vasquez, Lu Tang

**Affiliations:** Columbia University, New York City, New York, United States; Santa Clara University, Santa Clara, California, United States; University at Albany, Rensselaer, New York, United States; Texas A&M University, College Station, Texas, United States

## Abstract

**Introduction:**

As U.S.-based Latino populations disproportionately experience chronic disease and age earlier than their non-Latino white counterparts, attentiveness to knowledge gaps in health communication is critical to address health disparities among Latino older adults.

**Methods:**

We conducted a cross-sectional survey among U.S.-based Latinos ages 50-86 (N=274; 2021) that measured physical and mental health information seeking across four sources—TV/radio/print-media, Internet/social-media, family/friends, and healthcare providers. Linear regression models examined patterns of personal, family, and mental health factors on each information seeking outcome.

**Results:**

Foreign-born Latinos had lower odds of mental health info-seeking on Internet/social media (p< 0.05) than U.S.-born Latinos. Compared to higher educational attainment, low education reduced odds of physical/mental health info-seeking in all media sources (p< 0.05). Family mental health history vs none saw 3-5 times the odds of physical/mental health info-seeking to all sources (p< 0.01). Additionally, moderate/severe mental health vs mild/none was associated with 2-3 times the odds of physical/mental health info-seeking among family/friends and healthcare providers (p< 0.01). English media use increased odds of physical health info-seeking in TV/radio/print-media and family/friends (p< 0.05), while Spanish/English media use increased odds of mental health info-seeking to healthcare providers (p< 0.05).

**Conclusions:**

Latino older adults report diverse yet unique patterns in health information acquisition. Those with self/familial mental illness especially seek health information more often than Latinos without a lived experience. Findings demonstrate the need to apply culturally appropriate health communication strategies to address the needs of older Latino adults, with special focus on families coping with mental illnesses.

